# Toxin-Antitoxin Modules Are Pliable Switches Activated by Multiple Protease Pathways

**DOI:** 10.3390/toxins8070214

**Published:** 2016-07-09

**Authors:** Meenakumari Muthuramalingam, John C. White, Christina R. Bourne

**Affiliations:** Department of Chemistry and Biochemistry, University of Oklahoma, Norman, OK 73019, USA; meena85@ou.edu (M.M.); John.C.White-1@ou.edu (J.C.W.)

**Keywords:** toxin-antitoxin, phenotypic changes, persister cells, post-segregational killing, bacterial physiology, environmental adaptation, cellular proteases, protease adaptors

## Abstract

Toxin-antitoxin (TA) modules are bacterial regulatory switches that facilitate conflicting outcomes for cells by promoting a pro-survival phenotypic adaptation and/or by directly mediating cell death, all through the toxin activity upon degradation of antitoxin. Intensive study has revealed specific details of TA module functions, but significant gaps remain about the molecular details of activation via antitoxin degradation used by different bacteria and in different environments. This review summarizes the current state of knowledge about the interaction of antitoxins with cellular proteases Lon and ClpP to mediate TA module activation. An understanding of these processes can answer long-standing questions regarding stochastic versus specific activation of TA modules and provide insight into the potential for manipulation of TA modules to alter bacterial growth.

## 1. Introduction

Bacteria undergo complex adaptive metabolic changes to promote survival under stressful or harsh conditions. Toxin-antitoxin (TA) modules play crucial roles in bacterial immunity and in adaptation to the environment [1,2,3]. There is a growing interest in co-opting TA modules to control bacterial growth, but significant questions remain concerning the mechanisms of antitoxin degradation, which is required to promote adaptive changes [4,5,6]. Current estimates put the occurrence of TA modules at greater than 10,000 loci, a testament to the widespread nature of these genetic units [7,8,9]. There is also a striking overlap between the pathways inhibited by the toxin components and many essential cellular functions that comprise current antibiotic targets [9,10,11,12,13].

TA modules have been the topic of several excellent recent reviews, and these provide a historical background for the discovery and functions of TA modules, as well as highlighting the potential involvement of TA modules in bacterial persistence and chronic infections [5,14,15,16,17]. The review by Page et al. summarizes the mechanism used for control of TA module transcription called conditional cooperativity, as was previously elaborated by Loris et al. and others [15,18]. In this mechanism, altering the relative stoichiometry of toxin to antitoxin molecules dictates the level of transcriptional repression mediated by the antitoxin, as demonstrated for the TA families Doc/PhD, CcdAB, ParDE, RelBE, Kid/Kis, VapBC [15]. The review by Chan et al. details the DNA-binding activity of antitoxins used for transcriptional repression, and different DNA-binding motifs within the same TA family serve to further sub-divide the system classifications [14]. Finally, the review by Woods outlined the known signaling pathways leading to TA module activation and current efforts to combat the resulting phenotypic persistence in *E. coli* [5]. The current review will build from these recent publications and focus on the limited knowledge for specific molecular details of degradation of the antitoxin component by cellular proteases, resulting in TA module activation.

## 2. Activation and Phenotypic Outcomes of TA Modules

Canonical TA modules encode a bacterial poison (toxin) and a more labile antidote (antitoxin); cellular homeostasis is maintained only with replenishing supplies of antitoxin. The antitoxins from Type II TA modules are proteins that are degraded by cytoplasmic proteases, particularly the Lon protease [6,8,19]. In TA modules, the antitoxin typically mediates transcriptional self-repression by binding to an operator in the promoter region of its own operon. Degradation of the antitoxin relieves this transcriptional repression and results in increasing the production of the dicistronic transcript for antitoxin and toxin. Depletion of antitoxin, which is only known to occur by proteolytic degradation, lowers the ratio of antitoxin to toxin thereby altering the stoichiometry of TA complexes and resulting in conditional cooperativity [15,18]. Therefore, the degradation of antitoxin is the determining factor for TA module effects on the cell.

The toxin proteins, which can be classified by their shared cellular targets, affect many different and physiologically essential pathways. Some toxins, such as the RelE and VapC families, inhibit translation by degrading RNA, while other families target cellular replication or membrane integrity [1,20]. Among the most paradoxical aspects of TA modules is the broad conservation of structure despite disparate sequences and without correlation to the targeted cellular pathways [21]. This is exemplified by high structural similarity between the ParE gyrase-inhibiting toxins and the RelE mRNA-degrading toxins [22,23,24,25]. Additional modularity exists between toxin families and antitoxin families, resulting in frequent interchange of module components [1,24].

TA pairs are frequently found on pathogenicity islands or on exogenous plasmids harboring multi-drug resistance cassettes [26,27,28]. While plasmid-borne TA modules were initially discovered due to their activity in mediating post-segregational killing, chromosomal TA modules appear to be highly redundant and implicated in physiological adaptation responses [29,30]. The outcomes of TA module activation are varied, even within the same toxin family, with reports of some modules promoting bacterial cell death [31,32,33], particularly in a plasmid segregation model [34,35,36,37,38,39,40], and others strongly implicated in promoting survival through the formation of biofilms and persister populations, which are highly resistant to antibiotics [41,42,43,44,45,46,47,48,49]. Thus, the roles of TA modules within infectious etiology can be positive, resulting in death of the bacterial cell or loss of genetic material, or negative by resulting in increased resistance to therapeutics [50,51].

**Figure 1:**
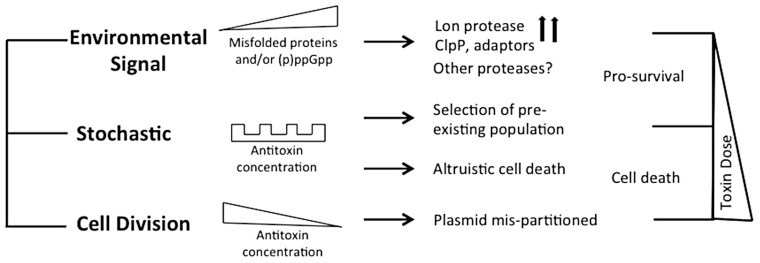
TA (Toxin-antitoxin) modules have been implicated in multiple models of bacterial physiology. Some pathways result in pro-survival changes, such as persister formation, or by selection of a stochastic population. Other changes result in cell death, consistent with the role of TA modules in the retention of genetic material. The toxin dose, or length of exposure, may also contribute to observed physiological changes.

The formation of antibiotic tolerant cells, including dormant or persister cells, has a dependence on TA modules presumably due to their ability to mediate reduced metabolic turnover [13,41,52,53,54,55,56]. The resulting dormant state is a heterogeneous population of phenotypically resistant cells [5,15,57,58]. Ectopic over-expression of RNA-degrading toxins, including RelE and MazF, can directly trigger the phenotypic changes resulting in persister cell formation [12,30,50,54,59,60,61]. It is still debated if a specific switch exists to activate given TA modules, or if the emergence of sub-populations results from stochastic selection of a pre-existing population with activated TA modules, or even if these models are conserved for all TA modules or are mutually exclusive (Figure 1) [30,41,45,46,47,62,63,64,65].

Populations of bacterial persisters have been characterized and recent work has found a direct link between the SOS and stringent response pathway leading to an up-regulation of polyphosphates through the Obg GTPase, and resulting in activation of TA modules in a protease-dependent manner [62,66,67]. In general, dormant persister populations present major impediments to the current antibiotic arsenal [36,68,69], and efforts are being made to re-awaken persisters with extrinsic signaling molecules [5,70] or to prevent their formation by blocking extracellular communication [71].

**Figure 2:**
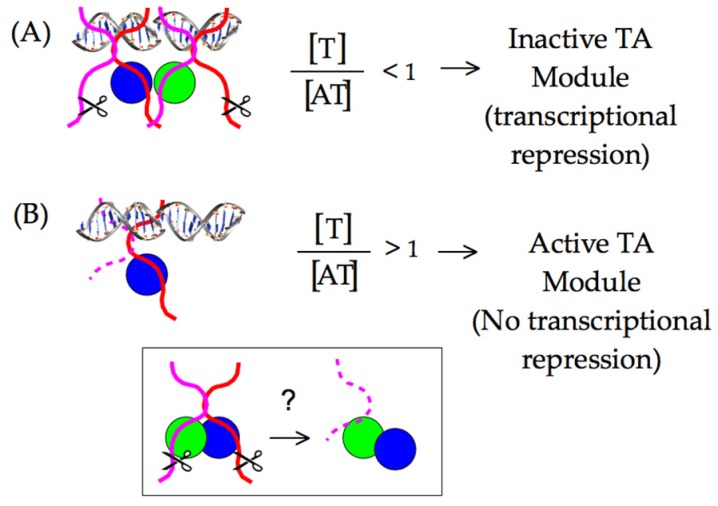
Activation of TA modules requires removal of antitoxin (red, magenta) from toxin (blue, green) by cellular proteases, and the stoichiometry of these two components control transcriptional repression through conditional cooperativity. (**A**) Normal cell growth typically has an excess of antitoxin modules, wherein transcription of the TA operon is repressed; (**B**) a TA module is activated both for transcription and for toxin activity in the cell by degradation of the antitoxin, shifting the stoichiometry to an excess of toxin molecules. It is not clear if degradation of TA complexes takes place directly from the DNA bound state, within the cytosol, or both. (Inset) The protease must be able to access the antitoxin, generally from the *C*-terminus, thereby removing it by overcoming typically very strong interactions with toxin.

Because degradation of the antitoxin relieves transcriptional repression by shifting the ratio of antitoxin to toxin (Figure 2), activation of TA modules can be measured by monitoring mRNA levels. This effect can be mimicked by inhibition of protein translation, which would prevent replenishment of antitoxin in response to basal turnover levels. Further, a decrease in mRNA level indicates a shift in the ratio of antitoxin to toxin (Figure 2). Any regulation of antitoxin degradation remains unknown, but one possible mechanism is by up-regulation of protease levels, such as noted for Lon during heat stress [72,73,74,75]. Several studies have demonstrated the up-regulation of TA modules under antibiotic or starvation conditions [76,77,78,79]. To analyze the expression levels of TA genes and the proteases Lon and ClpP under various antibiotic and stress conditions, RNA-Seq data files were downloaded from the NCBI database and analyzed according to published protocols, with results shown in Table 1 [80].

Within these data sets (Table 1), the *rnlBA* module was highly up-regulated in starvation conditions, as was *yafO-yafN*, *mqsAR*, *hicBA* and the *yafQ* and *cbtA* toxins. The up-regulation of a toxin gene without a similar up-regulation in antitoxin is surprising, and was also observed for the *mazF*, *yafQ* and *yoeB* toxins during heat shock treatment, while the antitoxin *rnlB* was highly increased relative to its toxin. In *Enterococcus* species, an increase in transcript levels of toxin mRNA levels (*relB*, *mazF*, and *higB*) during heat stress was noted while antitoxin transcripts remained constant [83], as also found for *mazF* and *relE* during heat shock conditions for strains of *E. coli* [84]. We surmise that these mRNAs may be enriched in sequences that are targeted for degradation, perhaps by up-regulated RNase toxins. In general, *yafO-yafN*, *yafQ-dinJ* and *yhaV-prlF* were the most up-regulated across these treatment conditions, wherein heat stress triggered an increase in RNA degrading toxins (*mazF*, *relE*, *yafQ*, *yafO*, *yoeB*, refer to Table 1). The only TA module transcripts observed to decrease in amounts were the *chpBS* and *fic-yhfG* during starvation and heat shock treatments. Other TA modules exhibited modest changes in transcript level, particularly during the ampicillin- and tetracycline-adapted treatments. Interestingly, the *hipBA* locus was not identified as up-regulated in any of these treatments.

The rifampicin treatment produced the largest responses of TA modules of any examined, which is contradictory as the treatment inhibits RNA polymerase. However, we note that the treatment was minimal, lasting only for 30 s prior to extraction. Further, strong upregulation of the *lon* protease gene was observed in these cells, and when translated to a protein product this could mediate efficient degradation of antitoxins. It is noteworthy that in this condition, up-regulation of *lon* levels was not sufficient to mediate degradation of antitoxins from the *cbtA-cbeA*, *fic-yhfG*, *higBA*, *hipBA*, and *mazEF* modules. Other than the rifampicin treatment, the increases in TA module transcripts arise due to degradation of the antitoxin without an increase in *lon* or *clpP* protease levels. Instead, it is more likely that the proteases have an increased enzymatic activity or a specific factor such as an adaptor is produced, as discussed below.

## 3. Proteases Shape the TA Module Proteome

TA modules affect cellular physiology following degradation of the antitoxin partner, thus freeing the active toxin component to interact with cellular pathways. Antitoxins are known to have very short half-lives of typically less than 15–20 min, while in *E. coli*, ~75% of the proteome has a half-life of 25 h [85,86]. The controlled half-life is mediated by cellular protease networks that recognize loosely structured *C*-terminal domains of the antitoxins [14,15,18]. This is an interesting paradigm in antitoxin activity, as the *C*-terminal domain interacts with the toxin partner frequently with a picomolar affinity, yet intrinsically disordered *C*-terminal free ends apparently can still be accessed for degradation (Figure 2) [87].

ATP-dependent proteases have been identified as mediators of this degradation in many situations, including the Lon (La) protease and the caseinolytic or caspase-3 like protease ClpP (see Table 2 and reviewed in [6,8,19,88,89,90]). Within the cellular environment, these ATP-dependent proteases act as regulators of protein quality control and can re-shape the proteome to mediate general adaptation responses [90,91,92], to DNA damage and metal exposure [93], to temperature changes [85,94] and for degradation of misfolded or aggregated proteins to promote optimal survival chances [95].

The Lon protease is found in all domains of life and has been described as having a “pivotal role in physiology” [85], although it is lacking in some Gram-positive bacteria, such as *Staphylococcus aureus* [96,97]. When present, Lon carries out the housekeeping functions in bacterial cells [88], with estimates of it carrying out ~50% of *E. coli* protein energy-dependent protein degradation [85]. Other studies with ClpP and its associated ATPase domain ClpX indicate the degradation up to 25% of the stress-induced SOS proteome, used as a means to turn off the SOS response [95].

The degradation signals for antitoxins or substrate specificities for proteases remain largely undefined. Advances in proteomic approaches have highlighted, in general, the context sensitivity and broad overlap of protease pathways [92]. In the simplest form, and seemingly the most likely to be used for antitoxins, are the preference of Lon for exposed hydrophobic patches [85,98,99] and for ClpXP, two *C*-terminal alanine residues [100]. Antitoxin sequences from the Toxin-Antitoxin DataBase (TADB) [101] were aligned and assessed in efforts to identify conserved potential protease “degrons” (Table 2). While the analysis was limited to the C-terminal portion, overall no significant consensus was observed outside of a series of small hydrophobic residues for the HicB antitoxin. The PhD antitoxin family has some conservation of hydrophobic residues in this region, which is interspersed with acidic residues. Similarly, the YefM antitoxin is enriched in acidic residues at its C-termini. The lack of conservation in sequences, even in patterns of hydrophobicity, is striking, particularly for families with many members, such as the RelB/DinJ antitoxins. The Epsilon antitoxin was degraded ~20 amino acids upstream of the *C*-terminus [102], which for most antitoxins would overlap with their toxin binding sites. It would seem that toxin binding would then hinder protease-mediated degradation, as was noted for the CcdA antitoxin [103].

Both Lon and Clp ATPases can recognize overlapping sequences present at the either termini of a client protein but with a commonality of being relatively unstructured, as is the case for at least some antitoxin proteins [15,18,125]. Further, recent studies have validated protease substrate “queuing”, where multiple client proteins can compete for degradation by Lon or ClpP, and the efficiency of degradation is then dictated by both relative affinity and concentrations [126]. The Lon and ClpP proteases have a common domain structure, with a typical ring-shaped oligomer contributing the protease domain and an additional ATPase domain in Lon or an associated protein with ClpP. For ClpP, this permits a modular function by pairing different Clp ATPase rings with the ClpP protease [127,128]. Both Lon and Clp systems undergo conformational changes that move loops found at the opening of the central pore in response to ATP hydrolysis and ADP release. These loops are typically lined with hydrophobic and especially aromatic ring-containing residues that result in a “pumping” action to unfold the client protein substrate and feed it into the associated protease chamber [127,128]. It is not clear if antitoxins are unfolded as part of their degradation, and this unfolded state would require the removal of the tightly bound toxin molecule.

The oligomeric state of the proteases affects its ability to degrade small or partially folded protein domains. Lon has been identified to form a concentration-dependent dodecamer (a dimer of hexamers) with a large central chamber that could accommodate small clients with residual secondary structure [129,130], such as perhaps an antitoxin molecule. Similarly, the central pore of ClpP is opened significantly when it associates with an ATPase oligomer [131]. Acyldepsipeptides (ADEPs), produced by *Streptomyces* species, can induce an opening of the central pore without an ATPase partner, resulting in constitutive activation and allowing the protease activity to access partially folded protein domains [132,133,134]. It is possible that ADEP’s are able to effectively eradicate persister cells [132,135], in part, by non-specific degradation of antitoxin molecules.

Of note, many antitoxins are no longer degraded when the gene for *E. coli* Lon or ClpP protease or *S. aureus* ClpP protease is deleted from the genome (Table 2). Antitoxins that have been identified as Lon substrates under different experimental conditions include PenI and PasA from low copy number plasmids and chromosomal *E. coli* RelB, ChpB, HicB and MazE during amino acid starvation (Table 2) [111,118,136,137,138]. The direct recognition and active unfolding of antitoxin molecules has not been demonstrated in vivo, and it remains unknown if the antitoxin can be degraded in the absence of unfolding. It is also unclear if the protease used for basal turnover during the “inactivated” state is the same as the one used for activation upon stress induction. Further, studies using in vivo protease deletion strains frequently result in reduction but not complete loss of antitoxin degradation, and in vitro assays with purified components are inherently slower (Table 2 and references therein). These data imply that the degradation pathway is more complex than simple association with Lon protease, and current models cannot capture the specificity measured for antitoxin activation under different adaptation conditions (for example, see Table 1).

What is clear is that not all TA modules are activated at the same time (for example, see Table 1). A stochastic mechanism would still require interaction of antitoxin with protease, but in an unregulated or “stochastic” way. This could give rise to specific TA module activation by selection during a stress, presumably based upon a positive effect mediated by the freed toxin. Another model is that sub-sets of antitoxins are specifically targeted in response to a stress, requiring controlled mechanisms for the protease and antitoxin interaction. Regardless of the activation model, it is feasible that the specificity of interactions between antitoxins and protease(s) are influenced by specific cellular conditions, including stressors [139], cell density [140], and protease concentration, particularly as induced by a heat shock condition [72,85,88,105,141,142]. Unfolded proteins allosterically activate the Mg^2+^ dependent Lon protease, during heat stress [99,131]. Its activity is also modulated by DNA binding and through interaction with polyphosphates, as produced during stringent response to amino acid starvation [143,144,145,146]. However, other studies have found no role for polyphosphates in Lon activation or in leading to a persistent phenotype in response to aminoglycosides [5,147,148]. Additionally, the antitoxin *prlF* was noted to have an activating effect on Lon via unknown mechanisms [149,150].

The degradation potential of antitoxins may also be impacted by the availability of adaptor proteins. The adaptor TrfA functions in the Gram-positive bacterium, *S. aureus*, which lacks a Lon protease, to deliver each of the three antitoxins (Axe1, Axe2 and a MazE) to the ClpCP complex [96,97]. Similarly, in *Caulobacter crescentus* the antitoxin SocA acts as an adaptor required for proteolytic degradation of toxin SocB [151]. This creates an inverted mechanism for the TA module and imparts a requirement of both the antitoxin and the ClpXP protease for growth of the organism. A related variation is a tripartite toxin-antitoxin-chaperone (TAC) module identified in *Mycobacterium tuberculosis* and encoding a SecB-like protein at the start of a HigBA operon. The SecB chaperone was demonstrated to protect the antitoxin HigA from aggregation and in so doing to facilitate its interaction with the HigB toxin [152,153]. While no adaptors have been identified for the Lon protease, there is no a priori reason to expect there are none [142]. A newly identified TA module, named GraTA, is degraded by a unique yet unidentified ATP-independent protease, and this module has also recently been demonstrated to target ribosome biogenesis in a unique mechanism [154,155]. This was the first report of antitoxin degradation independent of Lon or ClpP.

Within protease systems it is clear that one protein client can be a substrate for multiple proteases [100,156]. For example, in *E. coli* the MazE antitoxin is degraded by ClpAP in normal growth conditions [111] but becomes a substrate for Lon during amino acid starvation [142,157]. Similarly, the antitoxin DinJ is stabilized in *E. coli* by the absence of either Lon or ClpXP [108]. While ectopic expression of the Lon protease identifies its ability to degrade many antitoxins, caution is warranted because the cellular context is not retained in these experiments [92]. However, overexpression of Lon protease in *E. coli* did not result in RelB or MazE antitoxin degradation, despite RelB being a known target for Lon protease [107], while the YefM antitoxin was very efficiently degraded [106,142]. Later studies found that YefM was efficiently degraded by heat shock treatment, and the stress factor *rpoH* played a role in sustained activation [105]. The stoichiometry of the toxin-antitoxin complex can further complicate this type of degradation kinetics, as the complex may alter the protease-antitoxin interaction. Given the cooperative control in TA modules, the availability of antitoxin for degradation is another important factor in the activation pathway.

## 4. Conclusions

There is a strong connection linking TA module activation to reports of protease involvement in the paradoxical survival of cells upon treatment with antibiotics, such as DNA damaging quinolones in *E. coli* and *P. aeruginosa* [158,159]. Treatment with this class of antibiotics is known to induce an SOS response which up-regulates Lon expression, potentially permitting survival with the aid of TA module activation. An important question for future studies is: does the disordered structure, or specific sequences in the antitoxin, determine the course of degradation, or, does the cell control this by changing expression or allosteric activation of proteases? Based on information in Table 1, only some TA modules are activated under a given stress while in Table 2, it seems that given antitoxin classes do not possess an intrinsic identification sequence or degron. The activation of TA modules has been proposed as a promising new method to control bacterial growth [3,4,6,10,43,77,160,161,162]. Advances in antibacterial developments by taking advantage of these potential weak points in bacterial physiology can be realized as this antitoxin-protease networked response is delineated.

## Figures and Tables

**Table 1:**
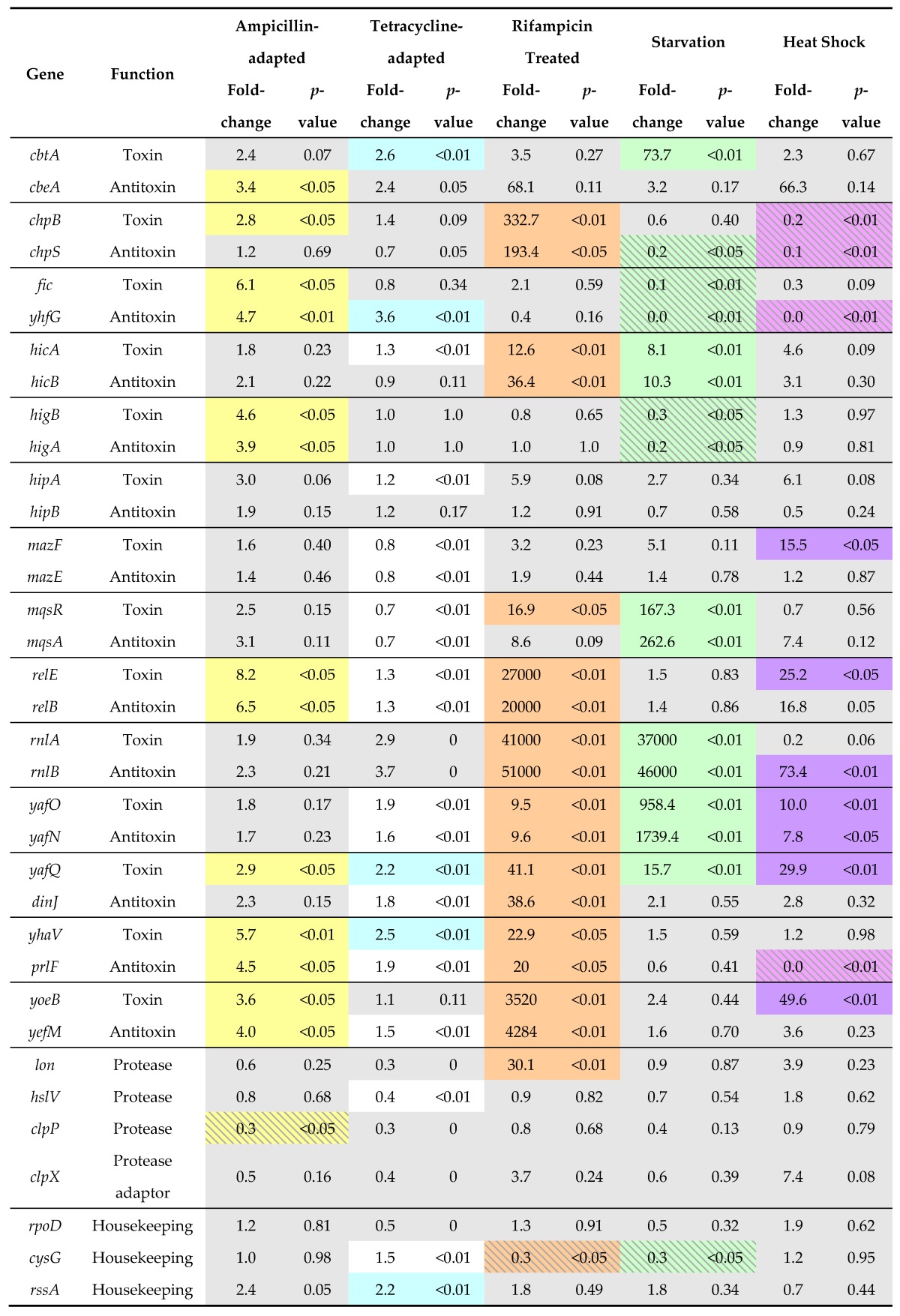
Transcript levels of TA (Toxin-antitoxin) modules in *E. coli* are altered in response to different treatment conditions.

Transcript levels, presented as a fold change versus untreated cultures, and *p*-values are given for the type II *E. coli* TA modules and the protease systems identified as mediating antitoxin degradation; housekeeping genes are included to demonstrate the level of variation between conditions. Values with *p*-values ≥0.5 are shaded grey to denote a lack of statistical significance within the datasets. Transcript amounts that were significantly up-regulated (>2-fold change) or down-regulated (<0.5-fold change) are shaded in colors. The *E. coli* str. K-12 substr. MG1655 genome was used as reference for alignment using BOWTIE 2.2.6 version [81]. To ensure specific alignment results, the parameter –k1 was set to report only the most distinct alignment per read with default settings. Differential expression analysis between control and various treatment samples were performed using edgeR tool kit [82]. All experiments used the MG1655 strain except the starvation experiments, which used *E. coli* strains TW11588 and IAI1. NCBI SRA identifiers for data sets analyzed: untreated population 1, SRX1561951; untreated population 2, SRX1561952; ampicillin, a cell wall inhibitor, adapted, SRX1561953; tetracycline, a translation inhibitor, adapted, SRX1561955; rifampicin, an RNA polymerase inhibitor, treated 0.5 min exponential phase, SRX682730; starvation in 48 h culture after 4 h of no flow, SRX1552242, SRX1552239, SRX1552245; heat shock 42 °C 10 min mid-log phase, SRX276081.

**Table 2:** Prevalence of different antitoxin families, proteases identified as mediating degradation, and *C*-terminal sequence conservation of antitoxin families.

TADB ^¥^	Pfam ^▯^	BToxDB ^❖^	TA Module	Protease	Consensus ^§^
Phd superfamily (PhD/YefM fold)					
27	2119	5	Antitoxin: PhD (Pfam02604) Toxin: Doc	ClpXP [37]	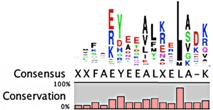
8	*N.D.*	4	Antitoxin: YefM Toxin: YoeB	Lon [104,105]	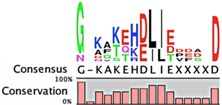
*N.D.*	*N.D.*	*none*	Antitoxin: Axe Toxin: Txe	ClpCP, adaptor TrfA [96,97]	*no sequence identified*
RelB Superfamily (Pfam 04221)					
214	666	16	Antitoxin: RelB Toxin: RelE	Lon [106,107]	No consensus for logo
			Antitoxin: DinJ Toxin: YafQ	Lon, ClpXP [108]	
PasA superfamily					
1	*N.D.*	*none*	Antitoxin: PasA Toxin: PasBC	Lon [109]	*only one sequence identified*
60	376	1	Antitoxin: ParD (Pfam 03693) Toxin: ParE	Lon [38]	No consensus for logo
VapB/MazE superfamily					
385	250	7	Antitoxin: VapB (Pfam 09957) Toxin: VapC	Lon [110]	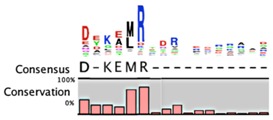
92	1960	9	Antitoxin: MazE (ChpB) (Pfam 04014) Toxin: MazF (ChpA)	ClpCP with TrfA; Lon, ClpAP [96,97,111,112]	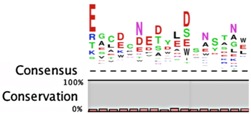
1	*N.D.*	2	Antitoxin: PemI/Kis Toxin: PemK/Kid	ClpAP [113]	*only one sequence identified*
112	*N.D.*	2	Antitoxin: HigA Toxin: HigB, ParE/RelE, HipA, GinD, Zeta	Lon [114]	No consensus for logo
1	*N.D.*	11	Antitoxin: HipB Toxin: HipA	Lon [115]	*only one sequence identified*
Unclassified antitoxins					
9	139	14	Antitoxin: CcdA (Pfam 07362) Toxin: CcdB	Lon [116]	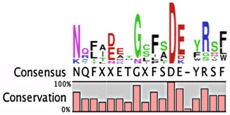
2	7	2	Antitoxin: Epsilon * (Pfam 08998) Toxin: Zeta	ClpXP [102]	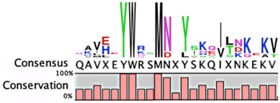
1	124	1	Antitoxin: MqsA (Pfam 15731) Toxin: MqsR	Lon, ClpXP [44,117]	Atypical antitoxin No consensus for logo
62	951	*none*	Antitoxin: HicB (Pfam 15919) Toxin: HicA	Lon [118]	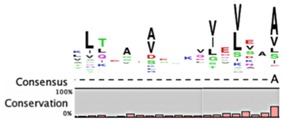
3	*N.D.*	*none*	Antitoxin: PrlF(MazE) Toxin: YhaV	unknown	*only three sequences identified*
1	*N.D.*	*none*	Antitoxin: MosA Toxin: MosT	unknown	*only one sequence identified*
2	*N.D.*	*none*	Antitoxin: YeeU Toxin: YeeV	unknown	*only two sequences identified*

Antitoxin grouping is based on Leplae et al. [119] and consistent with that found by Arbing et al. [120]. ¥ Toxin Antitoxin Database [101]; **▯** Number of entries in the Protein Families Database [121]; **❖** Database of toxin-antitoxin structural depositions database [122]; § Alignments were constructed using the ClustalW algorithm, and were visualized as a consensus sequence with the online server WebLogo [123] and with CLC Genomics Workbench© 8.0.3 [124]. *N.D.*, no data available. * Reported analysis of cleaved products of Epsilon antitoxin from in vitro assays revealed major cleavage sites ~20 amino acids from the *C*-terminal residue, and these sites were enriched for Leu, Asn, Glu and Val residues [102].
